# EMMPRIN contributes to the *in vitro* invasion of human salivary adenoid cystic carcinoma cells

**DOI:** 10.3892/or.2011.1606

**Published:** 2011-12-22

**Authors:** XINJIE YANG, PU ZHANG, QIN MA, LIANG KONG, YUAN LI, BAOLIN LIU, DELIN LEI

**Affiliations:** 1 Department of Oral and Maxillofacial Surgery, School of Stomatology, The Fourth Military Medical University, Xi’an 710032, P.R. China; 2 Department of Oral Pathology, School of Stomatology, The Fourth Military Medical University, Xi’an 710032, P.R. China

**Keywords:** salivary adenoid cystic carcinoma, invasion, extracellular matrix metalloproteinase inducer, matrix metalloproteinases

## Abstract

Extracellular matrix metalloproteinase inducer (EMMPRIN) is a transmembrane glycoprotein that is involved in tumor invasion by stimulating matrix metalloproteinase (MMP) expression. Our previous immunohistochemical study found that the expression of EMMPRIN in salivary adenoid cystic carcinoma (SACC) was positively correlated with tumor perineural and perivascular invasion. The present study was designed to further investigate the role of EMMPRIN in the invasion of SACC. Western blot results showed that EMMPRIN was upregulated in the highly metastatic SACC cell line SACC-LM, compared to SACC-83, a SACC cell line with low metastatic ability. Blocking of EMMPRIN by its antibody significantly decreased the adhesion, secretion of MMP-2 and MMP-9, and invasion activity of SACC-LM cells *in vitro* (P<0.01). Co-cultures of SACC-LM cells with fibroblasts significantly produced elevated levels of MMP-2 and MMP-9, and promoted the *in vitro* invasion activity of SACC-LM cells, compared with cultures of SACC-LM cells alone (P<0.01). These results indicate that EMMPRIN may play an important role in the invasion of SACC by stimulating the expression of MMP-2 and MMP-9 in tumor and stromal cells.

## Introduction

Salivary adenoid cystic carcinoma (SACC) is one of the most frequent malignancies of the salivary glands, constituting approximately 18% of all salivary gland malignancies (1). It has a protracted clinical course with diffuse invasion, local recurrences, late distant metastases, and poor response to classical chemotherapies (2). A prominent feature of SACC is its high affinity for basement membrane-rich tissues, such as nerves and blood vessels (3). The infiltration of major nerves and blood vessels in SACC could prevent complete surgical resection and lead to a worse prognosis (4,5).

The special proclivity of SACC cells to invade nerves and endothelial sheaths may be related to their high chemotactic response to the extracellular matrix (ECM) (3,6). Degradation of the ECM by matrix metalloproteinases (MMPs) is a crucial step in tumor invasion and metastasis (7). In many tumors, expression of MMPs is mainly regulated by tumor-stroma interactions via a tumor cell-associated protein and extracellular MMP inducer (EMMPRIN, CD147), which is a transmembrane glycoprotein that belongs to the immunoglobulin superfamily (8). EMMPRIN has been found to promote tumor invasion and metastasis by mediating the expression of MMPs within the tumor microenvironment (9,10). In our recent immunohistochemical study, we found that EMMPRIN expression in SACC was positively-associated with tumor perineural and perivascular invasion, and that MMP-2 and MMP-9 were expressed both in the tumor and stromal compartments (11). Thus, we hypothesized that EMMPRIN may be a key mediator in the invasion of SACC through functionally mediating the expression of MMPs in the surrounding stromal cells and tumor cells.

To examine our hypothesis, we evaluated the effects of blocking EMMPRIN by its antibody on human highly metastatic SACC cells (SACC-LM cells) when cultured alone or co-cultured with fibroblasts. In the present study, we performed an *in vitro* assay for invasive behavior using modified Boyden chambers to explore the role of EMMPRIN in the invasion of SACC. We also examined the tumor cell adhesion and MMPs expression activity to explore the possible mechanism of EMMPRIN in the invasion of SACC.

## Materials and methods

### Cell culture

Salivary adenoid cystic carcinoma cells with high metastatic potential (SACC-LM) and low metastatic potential (SACC-83) were provided by the College of Stomatology, Beijing University (12,13). Human embryonic pulmonary fibroblasts were purchased from the Chinese Academy of Medical Sciences. All cells were cultured in DMEM (Gibco, USA) supplemented with 10% FBS (Gibco) at 37°C in a humidified atmosphere of 5% CO_2_.

### Western blot analysis of EMMPRIN

The cellular proteins were separated by 10% SDS-PAGE and transferred onto PVDF membranes (Pall Corporation). Non-specific binding sites on the membrane were blocked in 5% skim milk for 1 h at room temperature. The membranes were then incubated with a 1:500 dilution of mouse anti-human EMMPRIN (Santa Cruz Biotechnology, Santa Cruz, CA, USA) or β-actin antibody (Sigma, USA) for 2 h at 37°C and followed by a reaction with goat anti-mouse IgG (Sigma) for 1 h at room temperature. After washing, the membranes were treated with enhanced chemiluminescence reagent (Santa Cruz Biotechnology) and exposed on Kodak X-ray film.

### Blockage of EMMPRIN

Tumor cells (1×10^6^) were collected in an Eppendorf tube containing 500 μl serum-free DMEM. The blockage of EMMPRIN was carried out with 5 μg monoclonal anti-EMMPRIN/CD147 antibody (Santa Cruz Biotechnology) under gentle agitation for 1 h. The cells were subsequently washed with PBS and used for further assay. As a control, the anti-EMMPRIN/CD147 antibody was replaced with PBS.

### Immunofluorescence and flow cytometry

Immunofluorescence and flow cytometry were performed to determine the blocking efficiency of anti-EMMPRIN/CD147 on EMMPRIN expression in tumor cells. Briefly, tumor cells of the EMMPRIN blockage group and control group were both stained with R-phycoerythrin (RPE)-labeled anti-CD147/EMMPRIN antibody (Serotec) for 30 min at 4°C. After washing with PBS, half of the cells were smeared on a slide and observed using immunofluorescence microscopy (Olympus), and the other half of the cells were subjected to flow cytometric analysis using a FACSCalibur flow cytometer and the CellQuest software (Becton-Dickinson).

### Adhesion assays

For adhesion assays of tumor cells to the ECM, the 96-well plates were coated with basement membrane Matrigel (BD Biosciences, USA) at a concentration of 5 mg/ml and incubated at 4°C overnight. Then, tumor cells (2×10^4^/well) suspended in serum-free DMEM were added to the wells and incubated at 37°C for 45 min. After removing the medium and non-attached cells, 0.2% crystal violet was added for 10 min and 5% SDS/50% ethanol was added for 20 min. Finally, the plate was read at 540 nm.

### Gelatin zymography analysis

Gelatin zymography was performed to assess the effect of EMMPRIN on gelatinase activity as previously described (14). Cells of each group were continuously cultured or co-cultured with equal fibroblasts in serum-free DMEM for 24 h. Conditioned medium was separated by SDS-PAGE under non-reducing conditions using 8% separating gel containing 0.1% gelatin (Sigma). The gels were incubated in a 2.5% Triton X-100 solution at room temperature with gentle agitation to remove SDS and then were soaked in reaction buffer (50 mmol/l Tris-HCl, 200 mmol/l NaCl, 10 mmol/l CaCl_2_, pH 7.5) at 37°C for 24 h. After reaction, the gels were stained for 6 h with staining solution and were destained for about 30 min. Gelatinolytic activity of MMPs was visualized as a clear band against a dark background of stained gelatin.

### In vitro invasion assay

The invasion activity of tumor cells *in vitro* were demonstrated in modified Boyden chambers (15). Transwell invasion chambers containing polycarbonate filters (pore size, 8 μm) were coated on the upper surface with basement membrane Matrigel (Becton-Dickinson). Tumor cells (1×10^5^) alone or together with equal numbers of fibroblasts in 200 μl serum-free DMEM were placed in the upper chamber. The lower chamber was filled with 600 μl conditioned medium (incubating fibroblasts in serum-free DMEM medium for 24 h) as chemoattractant. After 24-h incubation, the cells on the upper surface of the filter were removed with a cotton swab. The cells that had invaded the Matrigel and reached the lower surface of the filter were fixed in methanol, stained with H&E, and counted under a magnification of ×400. We chose five fields and counted the number of the invaded cells.

### Statistical analysis

All experiments were performed in triplicate and the data were calculated as means ± SD. Data analysis was performed by SPSS 16.0 software (USA). Multiple groups were analyzed by one-way ANOVA tests. Single group data was assessed using the Student’s t-test. P-values <0.05 were considered statistically significant.

## Results

### EMMPRIN expression in SACC-LM and SACC-83 cells

**Figure 1:**
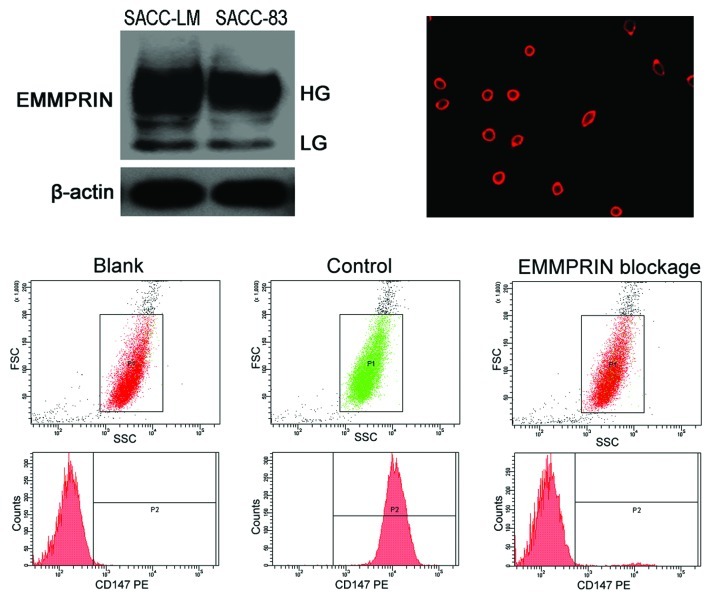
(A) The level of EMMPRIN protein was assessed by Western blotting. The expression of EMMPRIN protein was higher in SACC-LM cells compared to SACC-83 cells. (B) Photomicrograph of suspended SACC-LM cells labeled for EMMPRIN. This positive reaction demonstrates the specific binding of EMMPRIN to the membrane of SACC-LM cells (original magnification, ×200). (C) The competitive bindings of anti-EMMPRIN/CD147 antibody to EMMPRIN on SACC-LM cells were analyzed by flow cytometry. RPE-labeled anti-CD147/EMMPRIN antibody presented a binding rate of 0.70±0.38% to the blank control SACC-LM cells, a high positive binding rate of 95.87±2.25% to control SACC-LM cells, and a binding rate of 1.48±0.47% to EMMPRIN- blocked SACC-LM cells.

To compare the levels of EMMPRIN expression among two SACC cell lines, we utilized Western blot analysis and demonstrated that EMMPRIN expression was significantly increased in SACC-LM cells in comparison to SACC-83 cells (Fig. 1A). Thus, the SACC-LM cell line was chosen for further experiments.

### Blocking of EMMPRIN by its antibody in SACC-LM cells

We first observed positive immunolabeling of EMMPRIN mainly on the membrane of control SACC-LM cells (Fig. 1B), and no positive immunolabeling of EMMPRIN on EMMPRIN-blocked SACC-LM cells by immunofluorescence. Then, we evaluated the blocking efficiency of the anti-EMMPRIN/CD147 antibody by flow cytometry (Fig. 1C). RPE-labeled anti-CD147/EMMPRIN antibody presented a gated positive binding rate of 95.87±2.25% to control SACC-LM cells. However, after SACC-LM cells were incubated with anti-EMMPRIN/CD147 antibody, RPE-labeled anti-CD147/EMMPRIN antibody presented only a rate of 1.48±0.47% to EMMPRIN blocked SACC-LM cells, which were close to 0.70±0.38% of the blank control SACC-LM cells. These results suggested that the anti-EMMPRIN/CD147 antibody effectively bound to and blocked the EMMPRIN molecule on the membrane of SACC-LM cells with a relatively high affinity.

### Effect of EMMPRIN on the adhesion activity of SACC-LM cells to the ECM

**Figure 2:**
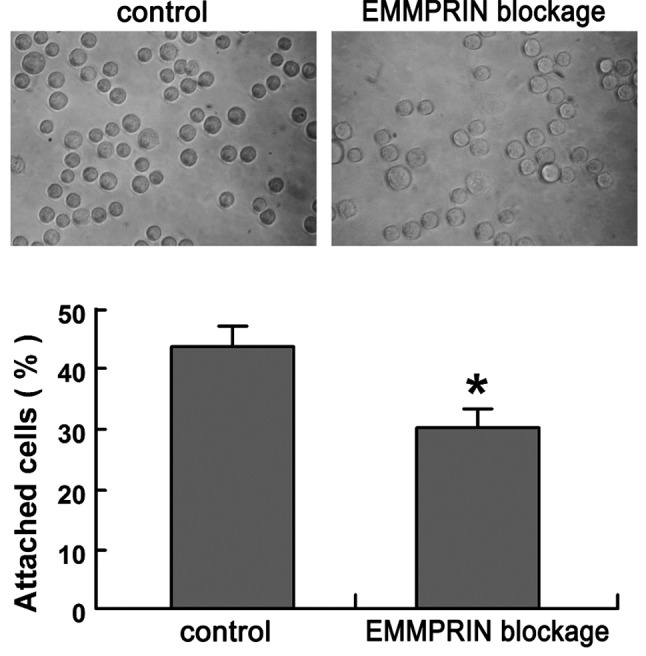
(A) Adhesion of SACC-LM cells and EMMPRIN-blocked SACC-LM cells to the Matrigel (original magnification, ×400). (B) The percentage of adhered tumor cells. ^*^P<0.01 compared to the control.

To examine the effects of EMMPRIN blockage on SACC-LM cell adhesion activity, adhesion assays of SACC-LM cells to the ECM were performed. After 45 min of incubation, a significant decrease in the amount of cells attached to the Matrigel-coated plates was observed in EMMPRIN blocked SACC-LM cells (30.26±2.86%), as compared to that in control SACC-LM cells (44.34±3.21%) (P<0.01; Fig. 2).

### Effect of EMMPRIN on gelatinase activity in SACC-LM cells

**Figure 3:**
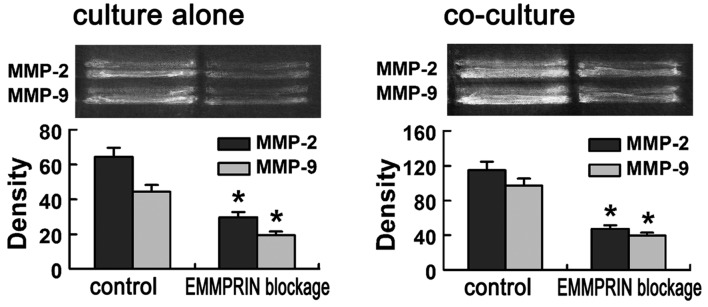
Gelatin zymography analysis of the MMPs secretion in SACC-LM cells and EMMPRIN-blocked SACC-LM cells when cultured alone (A) or co-cultured with fibroblasts (B). Top, representative images; bottom, gray scale analysises.^*^ P<0.01 compared to the respective control groups.

Since MMPs play critical roles in tumor cells invasion, we examined the effect of blocking EMMPRIN by its antibody on the enzyme activity of MMP-2 and MMP-9 using gelatin zymography. The gelatinase activity of both MMP-2 and MMP-9 were found to be reduced markedly in EMMPRIN blocked SACC-LM cells compared to that in control SACC-LM cells (P<0.01; Fig. 3A).

To mimic the *in vivo* tumor-stroma interaction in the local microenvironment, SACC-LM cells were co-cultured with (1:1) human fibroblasts and exhibited an enhanced gelatinase activity, that was 1.78±0.45-fold (MMP-2), and 2.35±0.53-fold (MMP-9) higher than that when cultured alone (Fig. 3). When co-cultured with human fibroblasts, the gelatinase activity of both MMP-2 and MMP-9 were still found to be reduced markedly in the EMMPRIN-blocked group compared with that in control group (P<0.01; Fig. 3B).

### In vitro invasion assay

**Figure 4:**
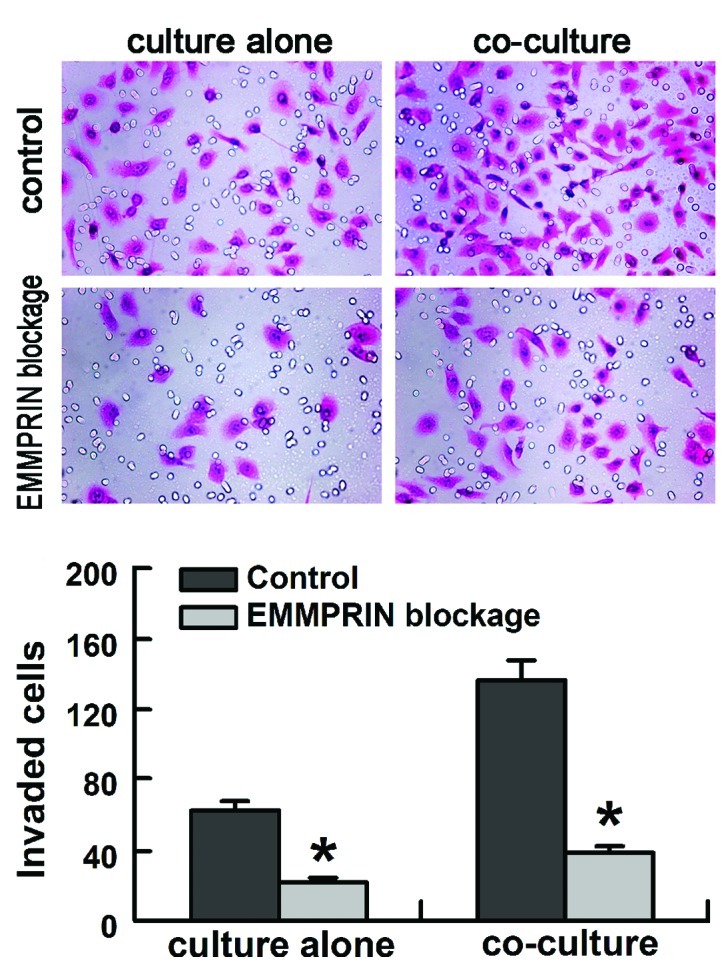
Effect of EMMPRIN on the *in vitro* invasion of SACC-LM cells. Matrigel invasion was measured in Boyden chamber assay. (A) SACC-LM cells and EMMPRIN-blocked SACC-LM cells that had invaded the Matrigel when cultured alone or co-cultured with fibroblasts (original magnification, ×400). (B) Quantitative analyses for the cells migrating through the Matrigel coated filter. ^*^P<0.01 compared to the control groups.

The role of EMMPRIN on the invasion activity of SACC-LM cells was evaluated *in vitro* using the modified Boyden chambers. The EMMPRIN-blocked SACC-LM cells exhibited much lower invasion activity compared with that in control SACC-LM cells when cultured alone or co-cultured with fibroblasts (Fig. 4A). When co-cultured with human fibroblasts, SACC-LM cells exhibited an enhanced invasion activity, that was 2.21±0.68-fold (SACC-LM cells group), and 1.71±0.54-fold (EMMPRIN-blocked SACC-LM cells group) higher than that when cultured alone (Fig. 4). Blocking EMMPRIN significantly inhibited the invasion activity of SACC-LM cells by 63.5±2.54% when cultured alone and by 71.6±2.83% when co-cultured with fibroblasts (P<0.01; Fig. 4B).

## Discussion

SACC is an invasive tumor with a special tendency to involve nerves and endothelial sheaths. EMMPRIN, also called CD147 or basigin, is a cellular adhesion molecule involved in cell-cell and cell-ECM interactions (8–10). EMMPRIN is highly expressed on the surface of various malignant tumor cells compared with their normal counterparts (16,17) and is involved in tumor invasion and metastasis (14,18). Our previous study found that overexpression of EMMPRIN was a prognostic factor for patients with SACC and was positively associated with the perineural and perivascular invasion of SACC (11). In the present study, we demonstrated that blocking EMMPRIN expression by its antibody could effectively inhibit SACC-LM cell adhesion and invasion activity *in vitro*.

Adhesion of tumor cells to surrounding tissues is the first step for tumor invasion and is highly dependent upon an increased production of proteases by the tumor cells. As an adhesion molecule, EMMPRIN has been reported to bind to a variety of cell types, including endothelial cells and fibroblasts, as well as the ECM (19). Matrigel is a basement membrane preparation containing almost all of the ECM components (20). The special proclivity of the SACC cells to invade basement membrane-rich tissues may be related to their high chemotactic response to the ECM (3,6). Our study showed that blocking of EMMPRIN could directly inhibit the attachment of SACC-LM cells to the Matrigel-coated plates. These results indicate that EMMPRIN induces the adhesion of the SACC-LM cells to the ECM.

Degradation of ECM are necessary steps in tumor local invasion (21). MMPs, a major family of enzymes that can degrade various components of the ECM, are believed to play critical roles in tumor invasion (7). Among MMPs, MMP-2 (gelatinase-A) and MMP-9 (gelatinase-B) are particularly upregulated in SACC and contribute to the invasion of tumor cells by degrading the ECM (22–24). Our previous study found that the expression of MMP-2 and MMP-9 was correlated with EMMPRIN expression in SACC (11). In the present study, we found that blocking of EMMPRIN expression in SACC-LM cells reduced the secretion of MMP-2 and MMP-9 when cultured alone or co-cultured with fibroblasts, thus inhibiting the invasion ability of SACC-LM cells through the reconstituted basement membrane *in vitro*. Our results suggest that EMMPRIN is involved in the invasion of SACC-LM cells by regulating the secretion of MMP-2 and MMP-9 to participate in the degradation of the ECM.

The search for MMP-inducing factors in tumor cells led to the identification of EMMPRIN, whose name reflects its activity. EMMPRIN has been found to facilitate tumor invasion and metastasis by regulating the expression of MMPs (8–11). EMMPRIN has also been found to promote tumor angiogenesis by stimulating the expression of MMPs and the vascular endothelial growth factor (VEGF) (25,26). Besides, EMMPRIN has been shown to promote tumor growth in an anchorage-dependent manner by inducing hyaluronan production (27), and to stimulate cancer cell proliferation via the activation of ERK1/2 and p38 MAPK signaling pathways (28). In addition, the latest studies showed that silencing of EMMPRIN gene expression via RNAi could inhibit the invasion activity of cancer cells (29,30). Our study suggested that blocking of EMMPRIN by its antibody could effectively inhibit the *in vitro* invasion activity of SACC-LM cells by inhibiting the expression of MMP-2 and MMP-9.

The interactions between tumor cells and the surrounding stromal cells have important implications in the invasion of many malignant tumors (31). Accumulating evidence suggests that EMMPRIN facilitates tumor invasion by participating in tumor-stroma interactions to stimulate the expression of MMPs in stromal cells (10,18). In this study, we found that co-culturing the SACC-LM cells and fibroblasts to mimic the tumor-stroma interactions produced elevated levels of MMP-2 and MMP-9 and gave rise to greatly increased *in vitro* invasion activity of SACC-LM cells, as compared with cultures of SACC-LM cells alone. We also found that blocking of EMMPRIN significantly inhibited the MMP-2 and MMP-9 secretion, and invasion activity of SACC-LM cells when cultured alone or co-cultured with fibroblasts *in vitro*. These results suggest that EMMPRIN participates in the tumor-stroma interactions by stimulating production of MMPs in both the tumor and stromal cells and thus facilitates the invasion of SACC-LM cells. Thus, further study of tumor-stroma interactions mediated by EMMPRIN may shed more light on the mechanism of SACC invasion.

In conclusion, this study showed that EMMPRIN played an important role in the invasion of SACC-LM cells through functionally mediating the expression of MMP-2 and MMP-9 both in tumor and stromal cells, and its antibody could effectively inhibit SACC-LM cells adhesion and invasion *in vitro*. Interruption of tumor-stroma interactions by blocking tumor EMMPRIN could be a target of anti-invasion therapy in SACC.

## References

[1] Li LJ, Li Y, Wen YM, Liu H, Zhao HW (2008). Clinical analysis of salivary gland tumor cases in West China in past 50 years. Oral Oncol.

[2] Fordice J, Kershaw C, El-Naggar A, Goepfert H (1999). Adenoid cystic carcinoma of the head and neck: predictors of morbidity and mortality. Arch Otolaryngol Head Neck Surg.

[3] Dardick I (1996). Color Attlas/Text of Salivary Gland Tumor Pathology.

[4] van der Wal JE, Snow GB, van der Waal I (1990). Intraoral adenoid cystic carcinoma. The presence of perineural spread in relation to site, size, local extension, and metastatic spread in 22 cases. Cancer.

[5] Barrett AW, Speight PM (2009). Perineural invasion in adenoid cystic carcinoma of the salivary glands: a valid prognostic indicator?. Oral Oncol.

[6] Shintani S, Alcalde RE, Matsumura T, Terakado N (1997). Extracellular matrices expression in invasion area of adenoid cystic carcinoma of salivary glands. Cancer Lett.

[7] Egeblad M, Werb Z (2002). New functions for the matrix metalloproteinases in cancer progression. Nat Rev Cancer.

[8] Toole BP (2003). Emmprin (CD147), a cell surface regulator of matrix metalloproteinase production and function. Curr Top Dev Biol.

[9] Sun J, Hemler ME (2001). Regulation of MMP-1 and MMP-2 production through CD147/extracellular matrix metalloproteinase inducer interactions. Cancer Res.

[10] Tang Y, Kesavan P, Nakada MT, Yan L (2004). Tumor-stroma interaction: positive feedback regulation of extracellular matrix metalloproteinase inducer (EMMPRIN) expression and matrix metalloproteinase-dependent generation of soluble EMMPRIN. Mol Cancer Res.

[11] Yang X, Dai J, Li T (2010). Expression of EMMPRIN in adenoid cystic carcinoma of salivary glands: correlation with tumor progression and patients’ prognosis. Oral Oncol.

[12] Li SL (1990). Establishment of a human cancer cell line from adenoid cystic carcinoma of the minor salivary gland. Zhonghua Kou Qiang Yi Xue Za Zhi.

[13] Dong L, Wang YX, Li SL (2011). TGF-beta1 promotes migration and invasion of salivary adenoid cystic carcinoma. J Dent Res.

[14] Quemener C, Gabison EE, Naïmi B (2007). Extracellular matrix metalloproteinase inducer up-regulates the urokinase-type plasminogen activator aystem promoting tumor cell invasion. Cancer Res.

[15] Chen W, Zhang HL, Jiang YG, Li JH, Sun MY (2009). Inhibition of CD146 gene expression via RNA interference reduces in vitro perineural invasion on ACC-M cell. J Oral Pathol Med.

[16] Zheng HC, Takahashi H, Murai Y (2006). Upregulated EMMPRIN/CD147 might contribute to growth and angiogenesis of gastric carcinoma: a good marker for local invasion and prognosis. Br J Cancer.

[17] Ueda K, Yamada K, Urashima M (2007). Association of extracellular matrix metalloproteinase inducer in endometrial carcinoma with patient outcomes and clinicopathogenesis using monoclonal antibody 12C3. Oncol Rep.

[18] Xu J, Xu HY, Zhang Q (2007). HAb18G/CD147 functions in invasion and metastasis of hepatocellular carcinoma. Mol Cancer Res.

[19] Stockinger H, Ebel T, Hansmann C, Kishimoto T, Kikutani H, von den Borne AEGKr (1997). EC/16/760 neurothelin/basigin/M6/EMMPRIN Workshop Panel Report. Leucocyte Typing VI: White Cell Differentiation Antigens.

[20] Kleinman HK, McGarvey ML, Hassell JR (1986). Basement membrane complexes with biological activity. Biochemistry.

[21] Liotta LA, Kohn EC (2001). The microenvironment of the tumor-host interface. Nature.

[22] Shirasuna K, Saka M, Hayashido Y, Yoshioka H, Sugiura T, Matsuya T (1993). Extracellular matrix production and degradation by adenoid cystic carcinoma cells: participation of plasminogen activator and its inhibitor in matrix degradation. Cancer Res.

[23] Vihinen P, Kähäri VM (2002). Matrix metalloproteinases in cancer: prognostic markers and therapeutic targets. Int J Cancer.

[24] Freitas VM, Vilas-Boas VF, Pimenta DC (2007). SIKVAV, a laminin alpha1-derived peptide, interacts with integrins and increases protease activity of a human salivary gland adenoid cystic carcinoma cell line through the ERK 1/2 signaling pathway. Am J Pathol.

[25] Tang Y, Nakada MT, Kesavan P (2005). Extracellular matrix metalloproteinase inducer stimulates tumor angiogenesis by elevating vascular endothelial cell growth factor and matrix metalloproteinases. Cancer Res.

[26] Tang Y, Nakada MT, Rafferty P (2006). Regulation of vascular endothelial growth factor expression by EMMPRIN via the PI3K-Akt signaling pathway. Mol Cancer Res.

[27] Marieb EA, Zoltan-Jones A, Li R (2004). Emmprin promotes anchorage-independent growth in human mammary carcinoma cells by stimulating hyaluronan production. Cancer Res.

[28] Li M, Zhai Q, Bharadwaj U (2006). Cyclophilin A is overexpressed in human pancreatic cancer cells and stimulates cell proliferation through CD147. Cancer.

[29] Chen X, Lin J, Kanekura T (2006). A small interfering CD147-targeting RNA inhibited the proliferation, invasiveness, and metastatic activity of malignant melanoma. Cancer Res.

[30] Zhu C, Pan Y, He B (2011). Inhibition of CD147 gene expression via RNA interference reduces tumor cell invasion, tumorigenicity and increases chemosensitivity to cisplatin in laryngeal carcinoma Hep2 cells. Oncol Rep.

[31] Bhowmick NA, Moses HL (2005). Tumor-stroma interactions. Curr Opin Genet Dev.

